# Gender Differences in the Correlates of Adolescents' Cannabis Use

**DOI:** 10.1080/10826080802238140

**Published:** 2008-08-20

**Authors:** Andrew W. Tu, Pamela A. Ratner, Joy L. Johnson

**Affiliations:** Nursing and Health Behaviour Research Unit, School of Nursing, University of British Columbia, Vancouver, British Columbia, Canada

**Keywords:** gender, adolescence, cannabis, marijuana, adolescent, marijuana use, risk factors, mental health, prevalence

## Abstract

Adolescents' gender-specific cannabis use rates and their correlates were examined. Data were obtained via a cross-sectional survey conducted in 2004 in British Columbia, Canada, funded by the Canadian Institutes of Health Research. School districts were invited to participate, and schools within consenting districts were recruited. In total, 8,225 students (50% male)from Grades 7 to 12 participated. About 73% were “White” and 47% had used cannabis in their lifetime. Cannabis users were grouped according to their frequency of use: “never users” “frequent users” or “heavy users” Male heavy cannabis users (14.3% of boys) were more likely to be in Grade 9 or higher; be Aboriginal; report poorer economic status; never feel like an outsider; frequently use alcohol and tobacco; and have lower satisfaction with family, friends, and school compared with boys that never used. Female heavy users (8.7% of girls) were more likely to be in a higher grade; report poorer economic status, mental health, and academic performance; frequently use alcohol and tobacco; and have lower satisfaction with their school compared with female never users. Three important gender differences in the multivariate analysis of the correlates of cannabis use were noted: school grade (for boys only), Aboriginal status (for boys only), and mental health (for girls only). Despite the limitations of relying on self-reports, a subset of youth appears to be at risk for excessive cannabis use that may impair life opportunities and health. The gender differences may be important in the design and implementation of prevention or treatment programs for adolescents.

Cannabis use is on the rise in Canada; recent data from the Canadian Community Health Survey (CCHS) indicate that cannabis use by Canadians aged 15 years and older almost doubled in just over a decade (Tjepkema, 2004). In 2002, 12.2% of Canadians reported using cannabis in the previous year, compared to 6.5% in 1989. There is regional variation in cannabis use rates, with the highest prevalence of 16% found in British Columbia (BC), Canada's westernmost province. Cannabis use is most prevalent among youth: 29% of 15- to 17-year-olds and 38% of 18- to 19-year-olds, in the CCHS, reported having tried it. In the 1998/99 Canadian National Longitudinal Study of Children and Youth, 9% of 13-year-olds, 25% of 14-year-olds, and 38% of 15-year-olds reported that they had used cannabis in the preceding year (Hotton and Haans, 2003). There is emerging evidence that the average age of initiation of cannabis use has declined in recent years, although the quality of these data is limited (Hall, Johnston, and Donnelly, 1999).

Although most youth who use cannabis do so only occasionally, there is a subset of youth that smoke it very frequently. According to the Ontario Student Drug Use Survey (OSDUS), between 1999 and 2003, there was a significant increase in the percentage of students in Grades 7 through 12 that reported *daily* cannabis smoking. In 2003, 4.2% of OSDUS participants reported daily cannabis use compared with 2.5% of participants in 1999 (Adlaf and Paglia, 2003). Daily use appears to be more common among boys rather than girls (6.2% of boys reported daily use compared to 2.2% of girls; Adlaf and Paglia). Higher rates of frequent use have been documented in BC, and they appear to be increasing. The McCreary Centre Adolescent Health Surveys of BC high-school students, which have been conducted about every 6 years, found that the percentages of 10- to 15+-year-old boys that smoked cannabis 20 or more times each month were 9% in 1992, 13% in 1998, and 18% in 2003. The percentages of female cannabis users who smoked 20 or more times each month were 4% in 1992, 6% in 1998, and 8% in 2003 (McCreary Centre Society, 1998, 2003). Population-based surveys of adolescents in Belgium have also found a gender difference in the prevalence rate and increasing rates over time (Kohn, Dramaix, Favresse, Kittel, and Piette, 2005; Kohn, Kittel, and Piette, 2004).

Several researchers have examined the factors associated with adolescents' cannabis use. Coffey, Lynskey, Wolfe, and Patton (2000) followed an Australian cohort of adolescents to obtain repeated measures of cannabis use. They found that correlates of cannabis use among students of about 15 years of age included having divorced or separated parents, peers that used cannabis, concomitant tobacco use, relatively heavy alcohol use, and antisocial behavior including property damage, interpersonal aggression, and stealing. Gender, which had a bivariate relationship with cannabis use (odds ratio = 1.4; 95% CI: 1.2–1.8), was eliminated from the multivariate model when these correlates were included. In a German cohort, von Sydow, Lieb, Pfister, Hofler, and Wittchen (2002) found that the significant predictors of “higher frequency” use included social-contextual factors such as parental death, deprived economic status, and the use of other illicit drugs. In a survey of Belgian adolescents, notable predictors of *monthly use* were being male; less educated; of non-Belgian nationality; tobacco, alcohol, or other illicit drug use; and moderate family integration (measured with items that assessed ease of talking with parents, whether the youths engaged in activities with their parents and in activities that had parental approval; Kohn, Kittel, and Piette, 2004). Predictors of *ever use* were being older; tobacco, alcohol, or illicit drug use; and stronger peer integration assessed as having close friends, being able to make new friends, and spending time with friends. In the UK, Miller and Plant examined the characteristics of 201 15 to 16-year-old “frequent users” of cannabis (Miller and Plant, 2002). Using cluster analysis of the youths' responses to questions about their demographics, substance use, families (e.g., parents' knowledge of the youth's whereabouts, rules, warmth, and support), friends (e.g., number of good friends, warmth, and mental support), leisure activities, and psychological status (e.g., self-esteem, aggression, and delinquency), three clusters emerged: a small group of mostly boys that was characterized by antisocial behaviour; a group that appeared to be “unhappy” (they had lower self-esteem, depression, and poorer parental and peer support); and a group that was characterized as “ordinary” (i.e., had good relationships with their family and friends, were obedient to society's rules, and displayed little antisocial behavior). Other observed associations with cannabis use include being Aboriginal (Novins and Mitchell, 1998), being peer integrated (Grunbaum, Tortolero, Weller, and Gingiss, 2000), truancy (Kohn, Dramaix, Favresse, Kittel, and Piette, 2005), lower academic performance (Resnicow, Smith, Harrison, and Drucker, 1999; Windle and Wiesner, 2004), poor physical health (Tims et al., 2002), and poor mental health (Patton et al., 2002; Rey, Martin, and Krabman, 2004), including depression (Degenhardt, Hall, and Lynskey, 2003; Patton et al.).

A gender difference in cannabis use has been identified although the nature of the difference has not been well explored. Previous studies that have noted gender differences have either done so within a specific population (Novins and Mitchell, 1998), without examining many correlates (Pape, Hammer, and Vaglum, 1994; Rodham, Hawton, Evans, and Weatherall, 2005), without stratifying their analysis by gender (Butters, 2005; Hofler et al., 1999; Kohn, Kittel, and Piette, 2004; Resnicow, Smith, Harrison, and Drucker, 1999; Swift, Hall, and Teesson, 2001), and without specifically focusing their analysis on cannabis use (Challier, Chau, Predine, Choquet, and Legras, 2000; Poulin, Hand, Boudreau, and Santor, 2005). The most consistent finding has been that boys are more likely to be “heavy users” than are girls (Kohn, Kittel, and Piette; Novins and Mitchell; Resnicow, Smith, Harrison, and Drucker). The factors associated with boys' and girls' use are not well described, and it is not known whether the risk factors differ by gender. This study aimed (a) to determine the gender-specific cannabis use rates of a large sample of adolescents from the province of BC, Canada, and (b) to determine factors associated with cannabis use and how those factors differ by frequency of use and gender. Based on the literature and what was possible with the available data set, we explored six factors of interest: sociodemographics, health status (physical and mental), life satisfaction, peer integration, academic performance, and other substance use.

## Methods

The data analyzed in this study were obtained via a cross-sectional survey focused on tobacco use, the *British Columbia Youth Survey on Smoking and Health 2* (BCYSOSH2), conducted between March and June 2004. As seen from the title, the survey questionnaire was constructed primarily to investigate youths' tobacco use and emerging tobacco dependence. In the past, youths advised our study team to ask about their cannabis use. The tobacco field has demonstrated that factors such as socioeconomic status, ethnicity, gender, and school performance are predictors of tobacco use, hence the inclusion of relevant indicators in the questionnaire.

To collect data from a large and diverse number of adolescent tobacco smokers, the majority of data was collected in regional school districts located outside the Greater Vancouver area (the largest city in the province). Of the 60 school districts in the province, 19 were contacted. Of the 19 school districts, 14 gave their schools permission to participate at their own discretion. Of the 86 schools in the 14 consenting school districts, 49 (57%) agreed to participate: 42 secondary schools, 5 alternative schools (designed for students not able to be successful in regular school environments), and 2 middle schools (with Grade 8 students).

The selection of students for inclusion varied across the 49 schools. The entire student body or all students in a particular grade were recruited in 22 schools, and the remaining 27 schools selectively recruited students. The nonrandom selection of students was typically carried out by attempting to include classes taken by most students (e.g., all students in the Grade 9 course, Career and Personal Planning). Two modes of questionnaire administration were offered to the schools, paper-based and Web-based formats. A passive consent procedure was employed; parents were informed about the study through letters carried home by the students. The recruitment and consent procedures and the questionnaire were approved by the relevant university and school district ethics review boards. To ensure that the participants benefited in some small way for their contribution to this research, we employed high school students to prepare school-level reports, which were posted on a website, so that the youths could see how their immediate peer groups compared with others in the Province.

Trained research personnel were present in every classroom to describe the purpose of the survey, to answer questions, and to take attendance. All research personnel were relatively young women with graduate level preparation in the social or health sciences, who had previous experience working with youth. Students were informed about the confidentiality of the survey and their rights as research participants, including their rights not to participate or to refrain from answering particular questions. The average response rate within each school was 84% with student absenteeism accounting for most of the non-response (the average refusal rate was less than 1%). About 3% of the surveys had to be destroyed mostly due to implausible responses. In total, 8,225 students completed the survey (6,544 paper-based and 1,681 web-based).

## Measures

### Response Variables

Table 1 provides the measures used in the study. The responses to two questions on cannabis use were used to create the dependent variable: “number of times used cannabis in entire life” (categorized as 0, 1–2, 3–9, 10–19, 20–39, 40–99, and 100+ times), and “number of times used cannabis in past 30 days” (0, 1–2, 3–9, 10–19, 20–39, 40+ times). We initially classified the participants into five groups: those who had never used cannabis (“never users”), those who had used cannabis but not in the last 30 days (“experimenters”), those who had used cannabis 1–2 times in the past 30 days (“occasional users”), those who had used cannabis 3–9 times in the past 30 days (“frequent users”), and those who had used cannabis 10+ times in the past 30 days (“heavy users”). The “experimenters” were found to be extremely heterogeneous in their amount of lifetime use and were omitted from these analyses. The “occasional” and “frequent” users were found to have similar associations and were therefore combined. Thus, the categories reported here are “never users,” “frequent users” (used cannabis 1–9 times in past 30 days), and “heavy users” (10+ times in past 30 days).

**Table 1:** Survey questions used in analysis

Area	Question	Responses
Response variables		
Cannabis use		
Lifetime use	*During your life*, how many times have you used marijuana (grass, pot, cannabis)?	0 times (never); 1 or 2 times; 3 to 9 times; 10 to 19 times; 20 to 39 times; 40 to 99 times; 100 or more times
Past 30 days	*During the past 30 days*, how many times have you used marijuana (grass, pot, cannabis)?	0 times; 1 or 2 times; 3 to 9 times; 10 to 19 times; 20 to 39 times; 40 or more times
Explanatory variables		
Sociodemographic factors		
Gender	Are you male or female?	Male; Female
Grade	What grade are you currently in?	Grade 8; Grade 9; Grade 10; Grade 11; Grade 12; Other (if other, please describe):
Ethnicity	How would you describe yourself? *Please mark all that apply* (these categories are from the 2001 Census)	Aboriginal/First Nation (e.g., North American Indian, Metis, Eskimo); Arab; Black (e.g., African, Haitian, Jamaican, Somali); Chinese; Filipino; Japanese; Korean; Latin American; South East Asian (e.g., Cambodian, Indonesian, Vietnamese, Laotian); South Asian (e.g., East Indian, Pakistani, Punjabi, Sri Lankan); West Asian (e.g., Afghan, Iranian) White/Caucasian; Other (specify)
Economic status	Compared to *other families in British Columbia*, is your household's financial situation (how much money your family has)…?	Much better; Somewhat better; About the same; Somewhat worse; Much worse
Health status		
Physical health	How would you rate your physical health?	Excellent; Very good; Good; Fair Poor
Mental health	How would you rate your emotional or mental health?	Excellent; Very good; Good; Fair Poor
Depression	12 items of the CES-D (Radloff, 1977)	
Life satisfaction	Multidimensional Students' Life Satisfaction Scale (40 items) (Huebner & Gilman, 2002)	
Academic performance	Compared to other students in your school, how do you rate yourself in the school work you do?	Far below average; Below average; Slightly below average; About average; Slight above average; Above average; Well above average
Peer integration	How often do you feel like an outsider (or left out of things at your school)?	All the time; Most of the time; Some of the time; Rarely; Never
Other substance use		
Cigarette use	About how many cigarettes have you smoked in your entire life?	I have never had a puff of a cigarette; I have only had a puff or a few puffs; 1–5 cigarettes; 6–15 cigarettes; 16–25 cigarettes; 26–99 cigarettes (fewer than 5 packs); More than 100 cigarettes (more than 5 packs)
Alcohol use	In the last 12 months, how often have you drunk alcohol – liquor (rum, whiskey, etc.), wine, beer, coolers?	I have never tried alcohol in my life; I have not had any alcohol within the last 12 months; I had a sip of alcohol to see what it was like; Drank only at special events (i.e., Christmas, weddings, etc.); Once a month or less often; 2 or 3 times a month; Once a week; 2 or 3 times a week; More than 3 times a week

### Explanatory Variables

*Sociodemographic* factors included gender, grade, ethnicity, and economic status. Because age and grade were highly correlated, only grade was entered into the model. Both had similar associations with the dependent variable; however, there was not a linear relationship between age and cannabis use. Ethnicity was categorized into three groups: White (European origin), Aboriginal, and other (visible minority status). There were not enough participants from other ethnic groups to create specific categories. Asian (35.1%) and South Asian (18.5%) youth comprised the two largest ethnic groups within the “other” category. Economic status was determined by asking the participants how they compared their family's financial situation with other families in BC (“much better,” “somewhat better,” “about the same,” “somewhat worse,” or “much worse”).

*Health status* was measured with two items and a scale. Each student rated his or her physical health and mental health on a 5-point ordinal scale from “excellent” to “poor.” Twelve of the 20 items of the Center for Epidemiological Studies Depression (CES-D) scale (Radloff, 1977) were used to assess depressive symptoms. Due to concerns about questionnaire length, only the 12 highest loading items from factor analysis were included in the survey. To develop cut points comparable to the full CES-D we created a possible total score of 60 by dividing the sum of the items by 12 and multiplying by 20. We categorized the severity of depressive symptoms by considering the common adult measure of ≥16 and the adolescent cut-off score of ≥24 (Roberts, Lewinsohn, and Seeley, 1991). We classified those with scores <16 as having minimal to no depressive symptoms, between 16 and 23 as having mild depressive symptoms, and ≥24 as having moderate to severe depressive symptoms. The CES-D has been shown to have good internal consistency and test-retest reliability with adolescent students, with results being comparable to those found in adult samples (Roberts, Andrews, Lewinsohn, and Hops, 1990).

*Life satisfaction* was measured with four facets of the Multidimensional Students' Life Satisfaction Scale (MSLSS): satisfaction with family, friends, school, and self (Huebner and Oilman, 2002). Previous studies using the MSLSS have reported high reliability and provided support for the dimensionality of the scale (Huebner and Gilman).

To assess *academic performance*, students were given a 7-point scale (“far above average,” “above average,” “slightly above average,” “average,” “slightly below average,” “below average,” or “far below average”) to rate their school work, compared with other students in their school.

*Peer integration* was determined by asking how often the participant felt like an outsider at school (“all the time,” “most of the time,” “some of the time,” “rarely,” or “never”).

*Other substance use* was determined from two questions: one about lifetime cigarette use (“none,” “one or a few puffs,” “1–5 cigarettes,” “6–15 cigarettes,” “16–99 cigarettes,” “more than 100 cigarettes”) and the other on alcohol consumption in past year (“none or one sip,” “only at special events,” “once a month or less,” “2 or 3 times a month,” “once a week,” or “more than once a week”).

## Data Analysis

Those with missing gender data were omitted from the analysis (*n* = 62). Differences between gender, sociodemographic, and cannabis use frequencies, were tested using chi-square or Mann-Whitney tests, where appropriate. Frequencies of the type of cannabis user (“never,” “frequent,” or “heavy”) stratified by the explanatory variables were produced by cross-tabulation for both genders; odds ratios were estimated with univariate multinomial logistic regression analysis. Multivariate multinomial logistic regression analysis was then performed to determine the associations between the levels of cannabis use with all significant explanatory variables from the crude analyses. Listwise deletion of participants with missing data led to a large percentage of students being deleted; therefore, multiple imputation was used to impute data. Those participants with missing data for more than 5 variables (*n* = 955) were omitted from the imputation procedure. The others had their missing information imputed using Schafer's NORM program (Schafer, 1999). Only the multivariate analysis was performed with the imputed data included. A *p* < 0.05 was considered statistically significant. SPSS version 12.0 (SPSS, Chicago, IL) was used for all analyses.

## Findings

Table 2 displays the sociodemographic characteristics of the participants, the amount of cannabis they used, and the age of initiation by gender. Our sample consists of equal numbers of boys and girls and relatively equal grade distributions from Grades 8 through 11, with a slight decline in numbers in Grade 12. The participants were predominantly white, which was expected given the sampling of the geographic areas and is consistent with census data. Many students did not report their family's economic status as being any worse than those in the rest of the Province; the boys more frequently reported that their economic status was better than did the girls.

**Table 2:** Selected sociodemographic and cannabis use frequencies by gender

	Boys (*n* = 4,064)		Girls (*n* = 4,099)		
			
Characteristic	Frequency	Percent	Frequency	Percent	*p*-value
Grade					<0.01
7–8	920	23.0	948	23.4	
9	808	20.2	761	18.8	
10	885	22.1	1,032	25.4	
11	858	21.5	845	20.8	
12	528	13.2	471	11.6	
Ethnicity					0.73
White	2,793	72.2	2,925	73.0	
Aboriginal	647	16.7	652	16.3	
Other	429	11.1	431	10.8	
Self-reported economic status of family compared with rest of province					<0.01
Better	1,393	39.3	1,291	34.8	
About the same	1,708	48.2	1961	52.8	
Worse	441	12.5	461	12.4	
Lifetime cannabis use					<0.01
0 times	1,966	51.9	2,069	52.9	
1–2 times	359	9.5	356	9.1	
3–9 times	257	6.8	407	10.4	
10–19 times	195	5.1	266	6.8	
20–39 times	195	5.1	254	6.5	
40–99 times	194	5.1	204	5.2	
100 or more times	624	16.5	356	9.1	
Cannabis use in past 30 days (of those who have used cannabis) (n = 3646)					<0.01
0 times	686	37.9	759	41.4	
1–2 times	314	17.3	423	23.1	
3–9 times	229	12.6	294	16.0	
10–19 times	153	8.4	135	7.4	
20–39 times	109	6.0	79	4.3	
40 or more times	320	17.7	142	7.8	
Age first used cannabis (*n* = 3638)					<0.01
< 9 years	157	8.7	53	3.5	
10 years	79	4.4	53	3.5	
11 years	119	6.6	79	5.2	
12 years	286	15.8	261	17.1	
13 years	406	22.5	208	13.6	
14 years	369	20.4	425	27.8	
15 years	245	13.6	295	19.3	
16 years	112	6.2	130	8.5	
≥ 17years	32	1.8	24	1.6	

About one-half of the sample (47.6%) indicated that they had tried cannabis in their lifetime and 28.9% reported that they used cannabis in the past 30 days. About the same percentage of boys and girls had used cannabis, but the girls tended to use it less frequently. Seventy-eight percent of the participants indicated that they first used cannabis between the ages of 12–15 years, which corresponds to Grades 7–10. The boys tended to initiate their use earlier than did the girls.

Tables 3 and 4 display the odds ratios obtained from the univariate multinomial logistic regression analyses for boys and girls, respectively. Stratifying our analysis by recruitment method (non-random vs. random) and data collection method (paper based vs. Web based), we found similar associations between cannabis use and all explanatory variables.

**Table 3:** Cross-tabulations and unadjusted odds ratios of type of cannabis user by sociodemographics, health status, academic performance, peer integration, other substance use, and life satisfaction for **boys only**

	Never used*(*n* = 1956)	Frequent user (*n* = 551)	OR (95% CI)†	Heavy user (*n* = 590)	OR (95% CI)†
Grade (no. (%))					
7–8‡	615 (31.6)	74 (13.7)	1.00	42 (7.2)	1.00
9	440 (22.6)	105 (19.4)	1.98 (1.44–2.74)	93 (16.0)	3.10 (2.11–4.55)
10	411 (21.1)	135 (24.9)	2.73 (2.00–3.72)	129 (22.2)	4.60 (3.18–6.65)
11	295 (15.1)	151 (27.9)	4.25 (3.12–5.81)	182 (31.3)	9.03 (6.28–12.99)
12	188 (9.6)	77 (14.2)	3.40 (2.38–4.87)	135 (23.2)	10.52 (7.17–15.42)
Ethnicity (no. (%))					
White‡	1428 (74.9)	387 (72.9)	1.00	375 (68.1)	1.00
Aboriginal	235 (12.3)	104 (19.6)	1.63 (1.26–2.11)	109 (19.8)	1.77 (1.37–2.28)
Other	244 (12.8)	40 (7.5)	0.61 (0.43–0.86)	67 (12.2)	1.05 (0.78–1.40)
Self-reported economic status of family compared with rest of BC (no. (%))					
Better‡	744 (40.5)	193 (37.2)	1.00	194 (37.9)	1.00
About the same	896 (48.7)	260 (50.1)	1.12 (0.91–1.38)	228 (44.5)	0.98 (0.79–1.21)
Worse	199 (10.8)	66 (12.7)	1.28 (0.93–1.76)	90 (17.6)	1.73 (1.29–2.33)
Physical health (no. (%))					
Excellent‡	729 (37.5)	188 (34.6)	1.00	143 (25.1)	1.00
Very good	717 (36.9)	197 (36.2)	1.07 (0.85–1.33)	192 (33.7)	1.37 (1.07–1.74)
Good	387 (19.9)	127 (23.3)	1.27 (0.98–1.65)	173 (30.4)	2.28 (1.77–2.94)
Fair/poor	112 (5.8)	32 (5.9)	1.11 (0.73–1.69)	61 (10.7)	2.78 (1.94–3.98)
Mental health (no. (%))					
Excellent‡	922 (48.1)	199 (37.3)	1.00	198 (36.1)	1.00
Very good	610 (31.8)	191 (35.8)	1.45 (1.16–1.81)	175 (31.9)	1.34 (1.06–1.68)
Good	274 (14.3)	97 (18.2)	1.64 (1.24–2.17)	115 (21.0)	1.95 (1.50–2.55)
Fair/poor	110 (5.7)	47 (8.8)	1.98 (1.36–2.88)	60 (10.9)	2.54 (1.79–3.60)
Depressive symptoms (no. (%))					
Minimal‡	1336 (75.5)	333 (67.1)	1.00	278 (61.2)	1.00
Mild	243 (13.7)	72 (14.5)	1.19 (0.89–1.59)	80 (17.6)	1.58 (1.19–2.10)
Moderate / severe	191 (10.8)	91 (18.3)	1.91 (1.45–2.52)	96 (21.1)	2.42 (1.83–3.19)
Academic performance compared to other students at school (no. (%))					
Above average‡	656 (36.7)	99 (19.8)	1.00	93 (19.3)	1.00
Slightly above average	390 (21.8)	75 (15.0)	1.77 (1.31–2.39)	77 (16.0)	1.82 (1.30–2.54)
About average	482 (27.0)	156 (31.2)	2.15 (1.62–2.83)	154 (32.0)	2.76 (2.05–3.72)
Slightly below average	154 (8.6)	104 (20.8)	3.23 (2.28–4.57)	82 (17.0)	4.32 (3.00–6.20)
Below average	105 (5.9)	99 (19.8)	4.17 (2.87–6.05)	76 (15.8)	7.65 (5.30–11.03)
Feel like an outsider at school (no. (%))					
Never‡	329 (18.8)	87 (18.0)	1.00	117 (25.3)	1.00
Rarely	778 (44.5)	230 (47.6)	1.12 (0.85–1.48)	187 (40.5)	0.68 (0.52–0.88)
Some of the time	443 (25.4)	109 (22.6)	0.93 (0.68–1.28)	88 (19.0)	0.56 (0.41–0.76)
All or most of the time	197 (11.3)	57 (11.8)	1.09 (0.75–1.60)	70 (15.2)	1.00 (0.71–1.41)
Number of drinks in past year (no. (%))					
None or one sip‡	1071 (57.0)	46 (8.7)	1.00	29 (5.1)	1.00
Drank only at special events	356 (19.0)	59 (11.2)	3.86 (2.58–5.78)	34 (6.0)	3.53 (2.12–5.87)
Once a month or less	273 (14.5)	139 (26.3)	11.86 (8.28–16.97)	83 (14.6)	11.23 (7.21–17.49)
2 or 3 times a month	102 (5.4)	149 (28.2)	34.01 (23.07–50.13)	150 (26.4)	54.31 (34.75–84.87)
Once a week	45 (2.4)	71 (13.4)	36.74 (22.82–59.13)	104 (18.3)	85.35 (51.34–141.89)
More than once a week	31 (1.7)	64 (12.1)	48.07 (28.56–80.90)	168 (29.6)	200.14 (117.59–340.65)
Number of cigarettes smoked in life (no. (%))					
None‡	1621 (84.2)	183 (34.1)	1.00	83 (14.4)	1.00
1 puff or few puffs	190 (9.9)	137 (25.5)	6.39 (4.89–8.35)	95 (16.5)	9.77 (7.02–13.59)
1–5 cigarettes	47 (2.4)	63 (11.7)	11.87 (7.90–17.85)	48 (8.3)	19.95 (12.61–31.56)
6–15 cigarettes	25 (1.3)	46 (8.6)	16.30 (9.78–27.15)	47 (8.2)	36.72 (21.55–62.56)
16–99 cigarettes	18 (0.9)	53 (9.9)	26.08 (14.96–45.48)	79 (13.7)	85.72 (49.09–149.67)
>100 cigarettes	24 (1.2)	55 (10.2)	20.30 (12.27–33.58)	224 (38.9)	182.28 (113.33–293.18)
Satisfaction with self (mean (*SD*))	4.78 (0.79)	4.80 (0.75)	1.04 (0.91–1.17)	4.68 (0.93)	0.87 (0.77–0.98)
Satisfaction with family (mean (*SD*))	4.60 (0.94)	4.17 (0.99)	0.64 (0.58–0.71)	3.99 (1.11)	0.55 (0.50–0.61)
Satisfaction with friends (mean (*SD*))	5.02 (0.77)	4.93 (0.81)	0.86 (0.76–0.97)	4.82 (0.90)	0.74 (0.65–0.83)
Satisfaction with school (mean (*SD*))	3.74 (1.02)	3.43 (0.94)	0.73 (0.66–0.81)	3.13 (0.99)	0.56 (0.51–0.62)

* Reference group for statistical comparisons.

† OR, unadjusted odds ratio; CI, confidence interval.

‡ Reference group.

*SD*, standard deviation.

**Table 4:** Cross-tabulations and unadjusted odds ratios of type of cannabis user by sociodemographics, health status, academic performance, peer integration, other substance use, and life satisfaction for **girls only**

	Never used* (*n* = 2064)	Frequent user (*n* = 722)	OR (95% CI)†	Heavy user (*n* = 358)	OR (95% CI)†
Grade (no. (%))					
7–8‡	616 (30.0)	135 (18.8)	1.00	35 (9.9)	1.00
9	427 (20.8)	121 (16.9)	1.29 (0.98–1.70)	57 (16.1)	2.35 (1.52–3.64)
10	518 (25.2)	217 (30.2)	1.91 (1.50–2.44)	100 (28.2)	3.40 (2.27–5.08)
11	322 (15.7)	164 (22.8)	2.32 (1.78–3.03)	98 (27.6)	5.36 (3.56–8.06)
12	169 (8.2)	81 (11.3)	2.19 (1.58–3.02)	65 (18.3)	6.77 (4.34–10.56)
Ethnicity (no. (%))					
White‡	1487 (73.4)	549 (76.9)	1.00	234 (66.7)	1.00
Aboriginal	238 (11.7)	120 (16.8)	1.37 (1.07–1.74)	94 (26.8)	2.51 (1.91–3.31)
Other	301 (14.9)	45 (6.3)	0.41 (0.29–0.56)	23 (6.6)	0.49 (0.31–0.76)
Self-reported economic status of family compared with rest of BC (no. (%))					
Better‡	753 (38.6)	200 (29.2)	1.00	98 (29.1)	1.00
About the same	1018 (52.2)	372 (54.3)	1.38 (1.13–1.67)	165 (49.0)	1.25 (0.95–1.63)
Worse	180 (9.2)	113 (16.5)	2.36 (1.78–3.13)	74 (22.0)	3.16 (2.24–4.45)
Physical health (no. (%))					
Excellent‡	517 (25.2)	103 (14.4)	1.00	24 (6.8)	1.00
Very good	874 (42.6)	271 (37.8)	1.56 (1.21–2.00)	84 (23.7)	2.07 (1.30–3.30)
Good	538 (26.2)	254 (35.4)	2.37 (1.83–3.07)	169 (47.7)	6.77 (4.34–10.55)
Fair/poor	124 (6.0)	89 (12.4)	3.60 (2.55–5.09)	77 (21.8)	13.38 (8.13–22.02)
Mental health (no. (%))					
Excellent‡	644 (31.8)	115 (16.2)	1.00	35 (9.9)	1.00
Very good	729 (36.0)	216 (30.5)	1.66 (1.29–2.13)	69 (19.4)	1.74 (1.14–2.65)
Good	447 (22.1)	209 (29.5)	2.62 (2.02–3.39)	118 (33.2)	4.86 (3.27–7.22)
Fair/poor	206 (10.2)	169 (23.8)	4.59 (3.46–6.10)	133 (37.5)	11.88 (7.93–17.79)
Depressive symptoms (no. (%))					
Minimal‡	1300 (65.7)	323 (46.4)	1.00	128 (37.8)	1.00
Mild	313 (15.8)	148 (21.3)	1.90 (1.51–2.40)	70 (20.6)	2.27 (1.66–3.12)
Moderate/severe	366 (18.5)	225 (32.3)	2.47 (2.01–3.04)	141 (41.6)	3.91 (3.00–5.11)
Academic performance compared to other students at school (no. (%))					
Above average‡	802 (41.8)	139 (21.1)	1.00	43 (13.1)	1.00
Slightly above average	459 (23.9)	131 (19.8)	1.65 (1.26–2.15)	62 (19.0)	2.52 (1.68–3.78)
About average	452 (23.6)	243 (36.8)	3.10 (2.44–3.94)	111 (33.9)	4.58 (3.16–6.63)
Slightly below average	116 (6.1)	86 (13.0)	4.28 (3.07–5.96)	59 (18.0)	9.49 (6.12–14.71)
Below average	88 (4.6)	61 (9.2)	4.00 (2.76–5.81)	52 (15.9)	11.02 (6.96–17.46)
Feel like an outsider at school (no. (%))					
Never‡	276 (14.6)	111 (17.1)	1.00	57 (17.9)	1.00
Rarely	902 (47.9)	305 (47.0)	0.84 (0.65–1.09)	129 (40.4)	0.69 (0.49–0.97)
Some of the time	507 (26.9)	161 (24.8)	0.79 (0.60–1.05)	84 (26.3)	0.80 (0.56–1.16)
All or most of the time	199 (10.6)	72 (11.1)	0.90 (0.64–1.27)	49 (15.4)	1.19 (0.78–1.82)
Number of drinks in past year (no. (%))					
None or one sip‡	1086 (55.2)	41 (5.9)	1.00	12 (3.5)	1.00
Drank only at special events	412 (20.9)	53 (7.6)	3.41 (2.23–5.20)	16 (4.6)	3.52 (1.65–7.49)
Once a month or less	304 (15.4)	196 (28.2)	17.08 (11.92–24.47)	50 (14.5)	14.89 (7.83–28.31)
2 or 3 times a month	127 (6.4)	219 (31.6)	45.68 (31.21–66.85)	99 (28.7)	70.55 (37.70–132.02)
Once a week	30 (1.5)	112 (16.1)	98.89 (59.41–164.59)	71 (20.6)	214.18 (105.18–436.17)
More than once a week	10 (0.5)	73 (10.5)	193.36 (93.11–401.54)	97 (28.1)	877.85 (369.80–2083.91)
Number of cigarettes smoked in life (no. (%))					
None‡	1690 (83.1)	170 (23.8)	1.00	20 (5.7)	1.00
1 puff or few puffs	209 (10.3)	163 (22.8)	7.75 (5.60–10.04)	44 (12.5)	17.79 (10.29–30.76)
1–5 cigarettes	59 (2.9)	104 (14.6)	17.52 (12.27–25.02)	34 (9.6)	48.70 (26.45–89.65)
6–15 cigarettes	29 (1.4)	73 (10.2)	25.02 (15.83–39.57)	28 (7.9)	81.59 (41.28–161.23)
16–99 cigarettes	30 (1.5)	88 (12.3)	29.16 (18.72–45.43)	68 (19.3)	191.53 (103.50–354.46)
>100 cigarettes	16 (0.8)	116 (16.2)	72.07 (41.75–124.42)	159 (45.0)	839.72 (426.61–1652.87)
Satisfaction with self (mean (*SD*))	4.76 (0.74)	4.64 (0.74)	0.82 (0.73–0.92)	4.60 (0.86)	0.76 (0.66–0.88)
Satisfaction with family (mean (*SD*))	4.57 (0.99)	3.92 (1.15)	0.58 (0.53–0.63)	3.74 (1.25)	0.51 (0.46–0.57)
Satisfaction with friends (mean (*SD*))	5.24 (0.70)	5.12 (0.76)	0.79 (0.71–0.89)	5.08 (0.85)	0.73 (0.63–0.85)
Satisfaction with school (mean (*SD*))	3.94 (0.94)	3.39 (0.93)	0.55 (0.50–0.60)	3.14 (1.01)	0.43 (0.38–0.48)

* Reference group for statistical comparisons.

† OR, unadjusted odds ratio; CI, confidence interval.

‡ Reference group.

*SD*, standard deviation.

Male *frequent* cannabis users tended to be in the higher grades, be Aboriginal, have poorer mental health, have moderate to severe depressive symptoms, report poorer academic performance, drink alcohol and smoke tobacco more frequently, and have lower satisfaction with their family, friends, and school compared with the boys who never used cannabis. In addition to these characteristics, male *heavy* users were more likely to have self-reported poorer economic status, poorer physical health, and lower satisfaction with themselves compared with the boys who never used cannabis. The “heavy users” were less likely to report rarely or sometimes feeling like an outsider at school compared with the boys who never used cannabis.

Female *frequent* cannabis users tended to be in the higher grades, be Aboriginal, have poorer economic status, be in poorer physical and mental health, have mild to severe depressive symptoms, report poorer academic performance, drink alcohol and smoke tobacco more frequently, and have lower satisfaction with their self, family, friends, and school compared with the girls who never used cannabis. Other ethnicity had a protective effect on cannabis use compared with self-identified “white” students. Female *heavy* users had the same associations *as frequent* female users, except that they were less likely to “rarely feel” like an outsider, compared with girls that never used cannabis.

The multivariate analysis is displayed in Table 5. After adjusting for all the explanatory variables, male *frequent* users were more likely to be in the higher grades, be Aboriginal, have poorer academic performance, drink alcohol and smoke tobacco more frequently, have higher self-satisfaction, and lower satisfaction with their family compared with the boys who never used cannabis. Male *heavy* users were more likely to be in the higher grades, be Aboriginal, report poorer economic status, never feel like an outsider at school, drink alcohol and smoke tobacco more frequently, and have lower satisfaction with their family, friends, and school compared with the boys who never used cannabis.

**Table 5:** Multivariate multinominal logistic regression for “frequent” and “heavy” users of cannabis versus “never used” by gender

	Boys				Girls			
		
	Frequent user*		Heavy user*		Frequent user*		Heavy user*	
				
	Odds ratio	95% CI†	Odds ratio	95% CI†	Odds ratio	95% CI†	Odds ratio	95% CI†
Grade								
7–8‡	1.00		1.00		1.00		1.00	
9	2.17	1.43–3.28	2.97	1.64–5.35	1.09	0.73–1.64	1.90	0.99–3.63
10	1.89	1.26–2.83	2.92	1.65–5.17	1.23	0.84–1.79	1.87	1.03–3.41
11	1.83	1.20–2.79	2.78	1.56–4.95	1.05	0.69–1.59	1.84	0.97–3.47
12	1.33	0.82–2.16	2.73	1.48–5.06	1.00	0.60–1.65	2.32	1.13–4.77
Ethnicity								
White‡	1.00		1.00		1.00		1.00	
Aboriginal	2.69	1.86–3.89	2.54	1.61–4.01	0.98	0.66–1.46	1.58	0.96–2.60
Other	0.63	0.40–1.01	1.00	0.60–1.64	0.40	0.25–0.66	0.54	0.28–1.07
Self-reported economic status of family compared with rest of BC Better‡	1.00		1.00		1.00		1.00	
About the same	1.18	0.89–1.56	1.17	0.83–1.64	1.23	0.92–1.65	1.03	0.68–1.57
Worse	1.39	0.90–2.16	2.09	1.26–3.47	2.19	1.41–3.40	2.56	1.42–4.61
Physical health Excellent‡	1.00		1.00		1.00		1.00	
Very good	0.98	0.72–1.34	1.22	0.83–1.79	0.93	0.64–1.34	0.88	0.46–1.66
Good	0.95	0.65–1.39	1.32	0.68–1.80	1.06	0.70–1.61	1.35	0.71–2.59
Fair/poor	0.66	0.36–1.24	1.19	0.60–2.45	1.05	0.59–1.88	1.23	0.54–2.80
Mental health								
Excellent‡	1.00		1.00		1.00		1.00	
Very good	1.26	0.92–1.72	1.22	0.83–1.79	1.31	0.89–1.93	1.49	0.80–2.77
Good	1.21	0.79–1.85	1.11	0.68–1.80	1.26	0.80–1.98	2.19	1.13–4.21
Fair/poor	1.59	0.86–2.96	1.21	0.60–2.45	1.82	1.04–3.18	3.53	1.64–7.60
Depressive symptoms								
Minimal‡	1.00		1.00		1.00		1.00	
Mild	0.82	0.55–1.21	0.94	0.58–1.53	1.44	0.98–2.09	1.27	0.75–2.13
Moderate/severe	1.25	0.79–1.98	1.01	0.59–1.73	0.98	0.65–1.49	0.91	0.52–1.61
Academic performance compared to other students at school								
Above average‡	1.00		1.00		1.00		1.00	
Slightly above average	1.22	0.84–1.78	1.05	0.65–1.70	1.30	0.89–1.90	1.97	1.10–3.52
About average	1.28	0.90–1.83	1.16	0.74–1.82	1.26	0.88–1.82	1.64	0.95–2.84
Slightly below average	1.73	1.10–2.73	1.69	0.98–2.92	1.58	0.94–2.66	2.58	1.29–5.18
Below average	1.64	0.98–2.74	1.59	0.87–2.88	1.15	0.63–2.08	1.71	0.79–3.69
Feel like an outsider at school								
Never‡	1.00		1.00		1.00		1.00	
Rarely	1.12	0.78–1.60	0.67	0.44–1.02	0.83	0.56–1.24	0.75	0.43–1.32
Some of the time	1.03	0.67–1.60	0.56	0.33–0.93	0.71	0.45–1.13	0.70	0.37–1.32
All or most of the time	0.96	0.55–1.68	0.48	0.25–0.90	0.54	0.29–1.01	0.51	0.22–1.15
Number of drinks in past year								
None or one sip‡	1.00		1.00		1.00		1.00	
Drank only at special events	3.06	1.94–4.84	2.49	1.25–4.99	3.12	1.90–5.12	2.81	1.10–7.15
Once a month or less	9.66	6.33–14.73	9.19	5.04–16.75	10.03	6.47–15.54	7.83	3.49–17.60
2 or 3 times a month	23.76	15.18–37.21	35.92	19.66–65.62	27.98	17.69–44.27	47.50	21.23–106.28
Once a week	17.50	9.98–30.68	37.26	18.80–73.87	46.43	25.10–85.91	91.82	36.94–228.22
More than once a week	25.75	13.93–47.61	84.05	40.87–172.86	68.62	28.54–165.00	216.37	72.59–644.97
Number of cigarettes smoked in life								
None‡	1.00		1.00		1.00		1.00	
One puff or a few puffs	3.74	2.69–5.19	5.54	3.64–8.44	3.95	0.89–17.47	12.04	6.35–22.84
1–5 cigarettes	7.56	4.65–12.30	12.26	6.88–21.83	9.45	6.04–14.78	24.58	11.86–50.96
6–15 cigarettes	8.12	4.30–15.31	18.55	9.41–36.55	12.18	6.76–21.91	33.80	14.73–77.60
16–99 cigarettes	12.30	6.23–24.29	35.35	17.30–72.21	18.39	10.12–33.42	85.70	38.78–189.39
More than 100 cigarettes	11.40	5.83–22.30	74.34	37.97–145.54	34.56	17.21–69.37	245.92	104.64–577.97
Satisfaction with self	1.53	1.20–1.95	1.26	0.95–1.66	1.28	1.01–1.62	1.35	0.98–1.85
Satisfaction with family	0.77	0.66–0.91	0.79	0.66–0.96	0.83	0.72–0.95	0.85	0.70–1.02
Satisfaction with friends	0.84	0.68–1.03	0.74	0.59–0.94	1.03	0.82–1.28	1.08	0.81–1.45
Satisfaction with school	0.96	0.82–1.11	0.80	0.66–0.97	0.86	0.73–1.02	0.79	0.64–0.99

* The referent is “never used.”

† CI, confidence interval.

‡ Reference group.

Female *frequent* users were more likely to report poorer economic status, poorer mental health, more frequent alcohol and tobacco consumption, being more satisfied with themselves, and being less satisfied with their family compared with the girls that never used cannabis. Girls who were categorized in the “other” ethnic category were less likely to be frequent cannabis users than were self-identified “white” girls. Female *heavy* users were more likely to report being in a higher grade, poorer economic status, poorer mental health, poorer academic performance, more frequent alcohol and tobacco consumption, and lower satisfaction with their school than did girls who never used cannabis.

## Conclusions

The prevalence of cannabis use found in this survey was much higher than that reported by the BC Adolescent Health Survey III (47.6% vs. 37.0%), which was carried out in 2003 by the McCreary Centre Society (2004), a nonprofit organization concerned with adolescent health. This was expected to some extent because we purposely sampled regions with higher than average rates of adolescent tobacco use. Nonetheless, the rate of use is higher than that reported in most, if not all, other countries (Korf, 2001; Vega et al., 2002). And, the rate of use in the previous month surpassed the rate of tobacco use over the same time period in this population (44.7% vs. 17.5%; Richardson et al., 2007). Of particular concern is the high number of “heavy users” (i.e., those who reported smoking cannabis 10+ times in the past month), particularly among boys (14.3%); a rate that has increased over the past 10 years (Tonkin, 2005).

The multivariate analysis revealed some striking gender differences in the correlates of both frequent and heavy cannabis use. In particular, grade is predictive of boys' frequent cannabis use, but not of girls'. This might suggest that girls are less influenced by the cannabis use of their peers or the social milieu established in school. Second, reporting relatively poor mental health placed girls at risk for frequent and heavy cannabis use, a factor not observed in the boys' model. Similarly, being Aboriginal placed boys at greater risk of frequent and heavy use of cannabis relative to White boys, a risk factor not observed among the girls. Aboriginal boys appear to be at “double risk” due to their Aboriginality and gender. Many researchers have observed that Aboriginal people in Canada live in circumstances that place them at risk for substance misuse. These conditions have arisen from the adverse sequelae of colonization, forced assimilation through residential schools where many were exposed to emotional, physical, and sexual abuse, and poverty. There is little work reported concerning ethnic and gender interactions in cannabis use, however a similar pattern has been noted among the Aboriginal communities of northern Australia (Clough et al., 2004).

A substance abuse^1^ problem among Aboriginals has been well documented in Canada (Health Canada, 1998, 1999; Statistics Canada, 1993). In 1997, the BC First Nations' Regional Health Survey (BCFNRH) reported that drug and alcohol issues were the primary health concerns among Aboriginal people in BC (First Nations Chiefs' Health Committee, 2000). The majority of participants of the BCFNRH reported that there had been no progress made in efforts to reduce the problem in the previous two years. From their Adolescent Health Survey III data, the McCreary Centre described the health status of Aboriginal youth (McCreary Centre Society, 2005). They reported that 53% of BC Aboriginal youth had ever used cannabis (compared with 36% of non-Aboriginal students). Among those that had tried cannabis, 36% used it 1–9 times in the past month and 25% used it 10+ times in the past month. Among the Aboriginal students that we surveyed, 40.8% had ever tried cannabis with 32.2% and 29.3% having tried it 1–9 times or 10+ times in the past month, respectively.

Self-reported economic status was found to be associated with cannabis use. Those who believed that their economic status was poorer than other families in BC were more likely to be frequent or “heavy users” (although not significant in frequent male users) compared with those who believed that their economic status was better than other families. The association between social-economic status (SES) and cannabis use has changed over time. Older prospective studies found that adolescents from families of higher SES were more at risk to use cannabis (Baumrind, 1985; Kandel, Kessler, and Margulies, 1978; Kaplan, Martin, and Robbins, 1985). The results of recent studies have been conflicting with some researchers reporting no association and others an inverse association between SES and cannabis use (Challier, Chau, Predine, Choquet, and Legras, 2000; Geckova, van Dijk, Groothoff, and Post, 2002; Kandel, Chen, Warner, Kessler, and Grant, 1997; Miller and Miller, 1997; von Sydow et al., 2002). This trend may be related to the decreasing cost of cannabis or the increasing ease of access.

Physical health was not associated with cannabis use. There was no discernable association between depressive symptoms and cannabis use. There have been conflicting reports about the association between depression and cannabis use (de Praia, Ruiz-Canela, and Martinez-Gonzalez, 2005). We found an association in the bivariate analyses, which may have been confounded with the more global assessment of mental health and gender.

Poor academic performance was associated with frequent use by boys and heavy use by girls, although the trend was inconsistent for the girls. Several studies have found that cannabis use increases the risk of poor academic performance and early school leaving (Fergusson, Horwood, and Beautrais, 2003; Lynskey and Hall, 2000; Miller and Miller, 1997). Fergusson, Horwood, and Beautrais suggested that cannabis use influences academic performance through social networks that produce attitudes or values that promote school failure or dropout. Our finding that those adolescents who reported feeling like outsiders at their schools were less likely to be cannabis users supports that theory (i.e., cannabis users develop “normative” social networks that share common values).

Other substance use proved to have the strongest association with cannabis use. The greater an adolescent's reported consumption of alcohol or tobacco the more likely he or she was to report being a “frequent” or “heavy user” of cannabis. This confirms the report from the McCreary Centre Society (2004) adolescent health survey that very few students use cannabis without using other substances.

In terms of life satisfaction, “frequent users” were more likely to be more satisfied with themselves and less satisfied with their families than were those who never used cannabis, regardless of gender. This may come about because cannabis use itself can lead to misperceptions about social cues, to protection from the social anxieties experienced by most youth, and to relatively strong group attachments with those engaged in similar behavior. Male “heavy users” are more likely to be less satisfied with their family and school than are boys who never use cannabis. These results support the findings of Butters who found that poorer family and school relations were associated with cannabis use (Butters, 2005).

There are some potential limitations to this study. First, the study relied on self-reported data; therefore, the responses may be biased. However, the survey was anonymous and in most cases, a large percentage of the school took part, so students should not have feared being identified by their responses. Second, we sampled from a population of adolescents with higher than average tobacco-smoking rates; therefore, comparisons can only be made with similar populations. Third, the small percentage of students that missed class the day the survey was administered was mostly truant. It has been reported that those who are truant have a higher risk of substance use (Hallfors, Cho, Brodish, Flewelling, and Khatapoush, 2006); therefore, the prevalence reports may be underestimated. However, our sample size and participation rate were large enough so that the effects would be minimal. Fourth, economic status and academic performance were assessed by the students' perceptions. In light of the limitations associated with self-reported academic performance, these findings should be viewed with caution. The association that we reported, however, may be underestimated because the validity of self-reported grades interacts with actual levels of school performance such that the poorer performers are likely to overestimate their performance (Kuncel, Crede, and Thomas, 2005). Less is known about the validity of adolescents' self-reported economic status. Frojd, Marttunen, Pelkonen, der Pahlen, and Kaltiala-Heino (2006) suggested that, although adolescents' perceptions of their family's economic status is likely associated with the actual status, they also may be indicative of the “psychological meaning” attached to the family's situation (i.e., financial problems) and could be considered a potential measure of risk independent of the actual status. Finally, we categorized the respondents into three categories: never, “frequent,” and “heavy users.” Although we combined various levels of use that had similar strengths of association with the hypothesized correlates, it may be that different levels of use or cut points yield different results.

The findings show important gender differences in predictors of “frequent” and “heavy cannabis use.” These differences may be important in the design and implementation of prevention or treatment programs for adolescents. For example, programs focussed on girls may need to address specifically mental health issues. Current adolescent substance abuse treatment programs have an average sustained abstinence rate of 32% at 12 months after treatment (Williams and Chang, 2000). Although there is evidence that treatment is better than no treatment, improvements in treatment can still be made. The effectiveness of prevention programs has been inconclusive. Two separate reviews have provided differing conclusions about the effect of school-based substance use prevention (Wiehe, Garrison, Christakis, Ebel, and Rivara, 2005; Skara and Sussman, 2003), which might be best suited for boys. Programs should be designed for each specific target population and not be seen as an all-encompassing tool.

In conclusion, the rates of cannabis use reported here, particularly excessive use, are troubling. Increasingly, youth appear to be turning to cannabis use while tobacco use is diminishing. Important gender differences in the prevalence of cannabis use, and its correlates, were noted. For example, mental health status was associated with girls' cannabis use and not boys'. The findings suggest that the uptake and use of cannabis may be very different for girls and boys. In addition, the correlates of “frequent” and “heavy use” differ, which suggests that excessive use may result for reasons other than ease of access and behavioral normalization. Further research is required to uncover the nature of these differences and to explicate their implications for intervention. Cannabis use has often been disregarded as a benign, youth-specific form of recreation. This may be the case for many if not most users; however, a subset of youth appears to be at risk for excessive use that may impair their life opportunities and health.

No conflicts of interest.

## Résumé

### Diferencias de género en los correlacionados del uso de canabis por los adolescentes

Les taux de consommation de cannabis spécifique au genre des adolescents, ainsi que leur mise en corrélation, ont été analysés. Les données ont été obtenues suite à une enquête transversale menée en Colombie-Britannique (Canada) en 2004, subventionnée par les Instituts de recherche en santé du Canada. Les arrondissements scolaires ont été invités à participer et les écoles sous la responsabilité des arrondissements consentants ont été recrutées. En tout, 8 225 élèves (50% mâles) des niveaux 7 à 12 ont participé. Environ 73% etaient “blancs” et 47% d'entre eux avaient déjà consommé du cannabis. Les utilisateurs de cannabis ont été divisés en groupes en fonction de la fréquence de leur consommation : “jamais”, “fréquente” et “très fréquente”. Les mâles du groupe de consommation *trèsfréquente* (14,3% des garçons), étaient majoritairement au Niveau 9 ou supérieur; ce groupe comprenait une plus forte proportion d'autochtones; les individus avaient un statut économique plus faible; ne se considéraient jamais comme des étrangers; consommaient fréquemment de l'alcool et du tabac; et avaient un taux de satisfaction plus faible envers leur famille, leurs amis et leur école, en comparaison aux gaçons quine consommaient pas. Les filles du groupe de consommation *trésfréquente* (8,7% des filles) étaient majoritairement aux niveaux scolaires plus élevés; avaient un statut économique plus faible, ainsi qu'une santé mentale plus fragile et des performances académiques plus faibles; consommaient fréquemment de l'alcool et du tabac; et avaient un taux de satisfaction plus faible envers l'école, en comparaison aux filles qui ne consommaient pas. Trois différences importantes entre les genres au cours de l'analyse à variables multiples des mises en corrélation de la consommation de cannabis ont été notees : les résultats scolaires (pour les garçons seulement), le statut d'autochtone (pour les garçons seulement) et la santé mentale (pour les filles seulement). Malgré les limites qu'impose l'auto-évaluation, un sous-groupe de jeunes semble être à risque de consommation excessive de cannabis qui peut compromettre leur avenir et leur santé. Ces différences entre les genres peuvent être importantes dans la conception et la mise en œuvre de programmes de prévention ou de traitements pour les adolescents.

## Resumen

### Différences spécifiques au genre et mise en corrélation relativement à; la consommation de cannabis chez les adolescents

Se examinaron los índices de uso de canabis entre los adolescentes, discriminadas por género y sus correlacionados. La informatión se recogió mediante un sondeo seccional conducido en 2004 en Columbia Británica, Canadá financiado por los Institutos Canadiense de Investigatión en Salud. Se invitó la participatión de los distritos escolares y se reclutaron escuelas dentro de aquellos distritos que habían consentido participar. En total, participaron 8.225 estudiantes (50% varones) del 7° al 12° grados. Alrededor del 73% eran de razablanca y un 47% había usado canabis en algún momento en su vida. Los usuarios se agruparon de acuerdo a la frecuencia de uso: “nunca”, “uso frecuente” o “uso intense”. Los que reportaron un uso *intenso* de canabis (el 14,3% de los varones) tenían mayor probabilidad de estar en el 9° grado o en un grado más alto; ser aborígenes; reportar una situatión económica más pobre; no sentirse nunca como extraños; consumir alcohol y tabaco con frecuencia; y tener un grado de satisfactión más bajo con la familia, las amistades y la escuela en comparación con los varones que nunca habían usado canabis. Las jóvenes adolescentes de uso *intenso* (8,7% de las mujeres) tenían mayor probabilidad de estar en uno de los grados más altos; reportar una conditión más pobre tanto económica como de salud mental y superación académica; uso frecuente de alcohol y tabaco; y tener un grado de satisfactión más bajo con la escuela en comparación con las mujeres que nunca usaron canabis. Se notaron tres importantes diferencias de género en el análisis multivariante de los correlacionados de canabis: el grado escolar (sólo para los varones), conditión de aborigen (sólo para los varones); y salud mental (sólo para las mujeres). A pesar de las limitaciones que existen cuando se depende de auto-informes, parece que hay un subconjunto de jóvenes que están en riesgo debido a un uso excesivo de canabis lo que puede reducir las oportunidades en su vida y perjudicar su salud. Las diferencias de género pueden ser importantes en el diseño e implementatión de programas de tratamiento para adolescentes.

## The Authors

**Andrew W. Tu, M.Sc.**, recently completed a Master of Science in Population and Public Health at Simon Fraser University. He is employed by the British Columbia Centre for Disease Control to pilot test an alcohol and drug use monitoring system for the Province. Previously, Mr. Tu was employed by University of British Columbia, School of Nursing as a data analyst. His current interests include epidemiology and biostatistics.

**Figure 1:**
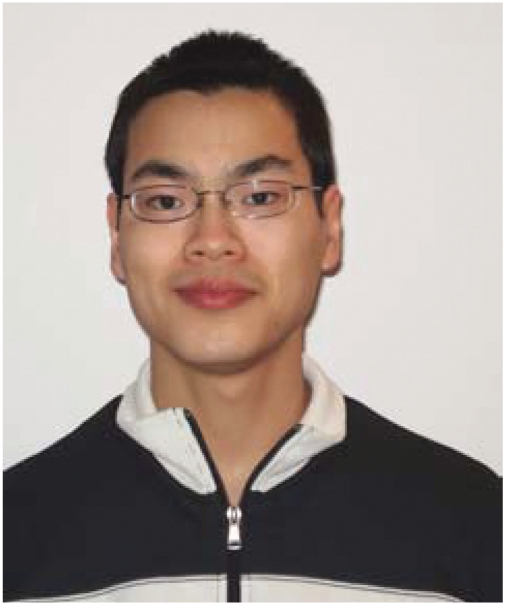


**Dr. Pamela A. Ratner** is a professor of nursing at the University of British Columbia and a Michael Smith Foundation for Health Research Senior Scholar. She is also co-Director of the Nursing and Health Behaviour Research Unit and of NEXUS, a multidisciplinary research unit focused on the social contexts of health behavior.

**Figure 2:**
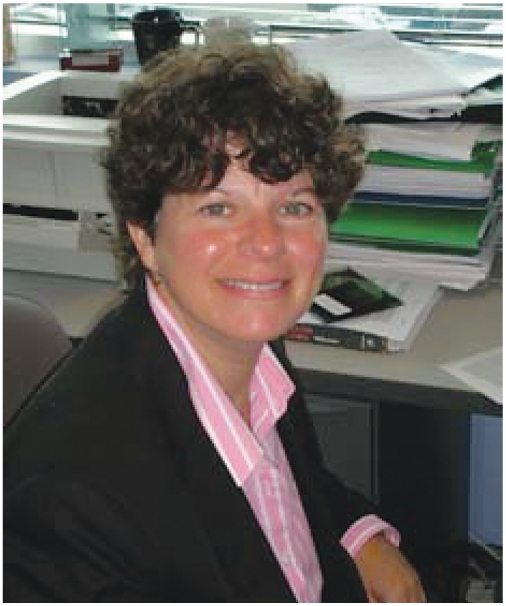


**Dr. Joy L. Johnson,** Professor in the School of Nursing at the University of British Columbia and a Canadian Institutes of Health Research Investigator, is also co-Director of the Nursing and Health Behaviour Research Unit and of NEXUS, a multidisciplinary research unit focused on the social contexts of health behavior.

**Figure 3:**
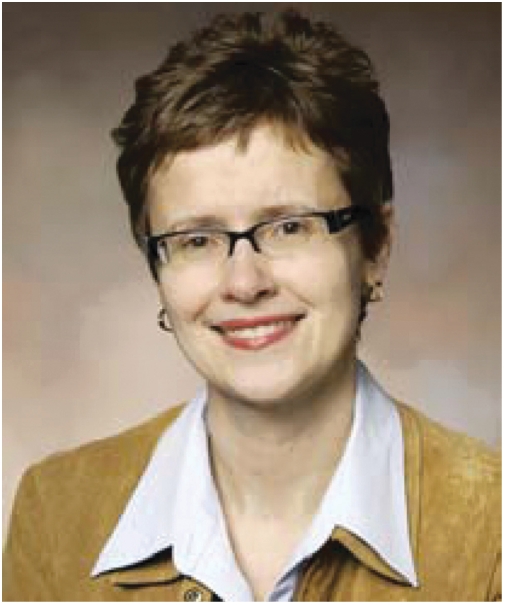


## Footnotes

1 The journal's style utilizes the category *substance abuse* as a diagnostic category. Substances are used or misused; living organisms are and can be *abused*. Editor's note.

## References

[b1] Adlaf E. M., Paglia A. (2003). Drug use among Ontario students, 1977–2003: Findings from OSDUS.

[b2] Baumrind D. (1985). Familial antecedents of adolescent drug use: A developmental perspective. NIDA Research Monograph.

[b3] Butters J. E. (2005). Promoting healthy choices: The importance of differentiating between ordinary and high risk cannabis use among high-school students. Substance Use & Misuse.

[b4] Challier B., Chau N., Predine R., Choquet M., Legras B. (2000). Associations of family environment and individual factors with tobacco, alcohol, and illicit drug use in adolescents. European Journal of Epidemiology.

[b5] Clough A. R., d'abbs P., Cairney S., Gray D., Maruff P., Parker R. (2004). Emerging patterns of cannabis and other substance use in Aboriginal communities in Arnhem Land, Northern Territory: A study of two communities. Drug and Alcohol Review.

[b6] Coffey C., Lynskey M., Wolfe R., Patton G. C. (2000). Initiation and progression of cannabis use in a population-based Australian adolescent longitudinal study. Addiction.

[b7] de Irala J., Ruiz-Canela M., Martinez-Gonzalez M. A. (2005). Causal relationship between cannabis use and psychotic symptoms or depression. Should we wait and see? A public health perspective. Medical Science International.

[b8] Degenhardt L., Hall W., Lynskey M. (2003). Exploring the association between cannabis use and depression. Addiction.

[b9] Fergusson D. M., Horwood L. J., Beautrais A. L. (2003). Cannabis and educational achievement. Addiction.

[b10] First Nations Chiefs' Health Committee (2000). Our nations on the edge of a new century: BC First Nations Regional Health Survey.

[b11] Frojd S., Marttunen M., Pelkonen M., der Pahlen B., Kaltiala-Heino R. (2006). Perceived financial difficulties and maladjustment outcomes in adolescence. European Journal of Public Health.

[b12] Geckova A., van Dijk J. P., Groothoff J. W., Post D. (2002). Socio-economic differences in health risk behaviour and attitudes towards health risk behaviour among Slovak adolescents. Sozialund Präventivmedizin.

[b13] Grunbaum J. A., Tortolero S., Weller N., Gingiss P. (2000). Cultural, social, and intrapersonal factors associated with substance use among alternative high school students. Addictive Behaviors.

[b14] Hall W., Johnston L., Donnelly N., H. Kalant, W. Corrigall, W. Hall, R. Smart (1999). Epidemiology of cannabis use and its consequences. The health effects of cannabis.

[b15] Hallfors D., Cho H., Brodish P. H., Flewelling R., Khatapoush S. (2006). Identifying High School Students “at Risk” For Substance Use And Other Behavioral Problems: Implications For Prevention. Substance Use & Misuse.

[b16] Health Canada (1998). National Native Alcohol and Drug Abuse Program (NNADAP) General Review.

[b17] Health Canada (1999). A second diagnostic on the health of Canada's First Nations and Inuit people.

[b18] Hofler M., Lieb R., Perkonigg A., Schuster P., Sonntag H., Wittchen H. U. (1999). Covariates of cannabis use progression in a representative population sample of adolescents: A prospective examination of vulnerability and risk factors. Addiction.

[b19] Hotton T., Haans D. (2003). Alcohol and drug use in early adolescence. Health Reports.

[b20] Huebner E. S., Gilman R. (2002). An introduction to the Multidimensional Students' Life Satisfaction Scale. Social Indicators Research.

[b21] Kandel D. B., Kessler R. C., Margulies R. Z., D. B. Kandel (1978). Antecedents of adolescent initiation into stages of drug use: A developmental analysis. Longitudinal research in drug use: Empirical findings and methodological issues.

[b22] Kandel D., Chen K., Warner L. A., Kessler R. C., Grant B. (1997). Prevalence and demographic correlates of symptoms of last year dependence on alcohol, nicotine, marijuana and cocaine in the U.S. population. Drug and Alcohol Dependence.

[b23] Kaplan H. B., Martin S. S., Robbins C., J. R. Greenley (1985). Toward an explanation of increased involvement in illicit drug use: Application of a general theory of deviant behavior. Research in community and mental health.

[b24] Kohn L., Dramaix M., Favresse D., Kittel F., Piette D. (2005). Trends in cannabis use and its determinants among teenagers in the French-speaking community of Belgium. Revue d'Epidémiologie et de Santé Publique.

[b25] Kohn L., Kittel F., Piette D. (2004). Peer, family integration and other determinants of cannabis use among teenagers. International Journal of Adolescent Medicine and Health.

[b26] Korf D. J. (2001). Trends and patterns in cannabis use in the Netherlands. Hearing of the Special Committee on Illegal Drugs.

[b27] Kuncel N. R., Crede M., Thomas L. L. (2005). The validity of self-reported grade point averages, class ranks, and test scores: A meta-analysis and review of the literature. Review of Educational Research.

[b28] Lynskey M., Hall W. (2000). The effects of adolescent cannabis use on educational attainment: A review. Addiction.

[b29] McCreary Centre Society (1998). Adolescent Health Survey II fact sheet: Marijuana use among BC youth.

[b30] McCreary Centre Society (2003). Adolescent Health Survey III fact sheet: Marijuana use among BC youth.

[b31] McCreary Centre Society (2004). Healthy youth development: Highlights from the 2003 Adolescent Health Survey.

[b32] McCreary Centre Society (2005). Raven's Children II: Aboriginal youth health in BC.

[b33] Miller D. S., Miller T. Q. (1997). A test of socioeconomic status as a predictor of initial marijuana use. Addictive Behaviors.

[b34] Miller P., Plant M. (2002). Heavy cannabis use among UK teenagers: An exploration. Drug and Alcohol Dependence.

[b35] Novins D. K., Mitchell C. M. (1998). Factors associated with marijuana use among American Indian adolescents. Addiction.

[b36] Pape H., Hammer T., Vaglum P. (1994). Are “traditional” sex differences less conspicuous in young cannabis users than in other young people?. Journal of Psychoactive Drugs.

[b37] Patten G. C., Coffey C., Carlin J. B., Degenhardt L., Lynskey M., Hall W. (2002). Cannabis use and mental health in young people: Cohort study. BMJ.

[b38] Poulin C., Hand D., Boudreau B., Santor D. (2005). Gender differences in the association between substance use and elevated depressive symptoms in a general adolescent population. Addiction.

[b39] Radloff L. S. (1977). The CES-D scale: A self-report depression scale for research in the general population. Applied Psychological Measurement.

[b40] Resnicow K., Smith M., Harrison L., Drucker E. (1999). Correlates of occasional cigarette and marijuana use: Are teens harm reducing?. Addictive Behaviors.

[b41] Rey J. M., Martin A., Krabman P. (2004). Is the party over? Cannabis and juvenile psychiatric disorder: The past 10 years. Journal of the American Academy of Child and Adolescent Psychiatry.

[b42] Richardson C. G., Johnson J. L., Ratner P. A., Zumbo B. D., Bottorff J. L., Shoveller J. A. (2007). Validation of the dimensions of Tobacco Dependence Scale for adolescents. Addictive Behaviors.

[b43] Roberts R. E., Andrews J. A., Lewinsohn P. M., Hops H. (1990). Assessment of depression in adolescents using the Center for Epidemiologie Studies Depression Scale. Psychological Assessment.

[b44] Roberts R. E., Lewinsohn P. M., Seeley J. R. (1991). Screening for adolescent depression: A comparison of depression scales. Journal of the American Academy of Child & Adolescent Psychiatry.

[b45] Rodham K., Hawton K., Evans E., Weatherall R. (2005). Ethnic and gender differences in drinking, smoking and drug taking among adolescents in England: A self-report school-based survey of 15 and 16 year olds. Journal of Adolescence.

[b46] Schafer J. L. (1999). NORM: multiple imputation of incomplete multivariate data under a normal model, version 2.03 [Computer software].

[b47] Skara S., Sussman S. (2003). A review of 25 long-term adolescent tobacco and other drug use prevention program evaluations. Preventive Medicine.

[b48] Statistics Canada (1993). 1991 Aboriginal Peoples Survey: Language, tradition, health, lifestyle and social issues.

[b49] Swift W., Hall W., Teesson M. (2001). Cannabis use and dependence among Australian adults: Results from the National Survey of Mental Health and Wellbeing. Addiction.

[b50] Tims F. M., Dennis M. L., Hamilton N., Buchan J., Diamond G., Funk R. (2002). Characteristics and problems of 600 adolescent cannabis abusers in outpatient treatment. Addiction.

[b51] Tjepkema M. (2004). Use of cannabis and other illicit drugs. Health Reports.

[b52] Tonkin R. S. (2005). British Columbia youth health trends: A retrospective, 1992–2003.

[b53] Vega W. A., Aguilar-Gaxiola S., Andrade L., Bijl R., Borges G., Caraveo-Anduaga J. J. (2002). Prevalence and age of onset for drug use in seven international sites: Results from the international consortium of psychiatric epidemiology. Drug and Alcohol Dependence.

[b54] von Sydow K., Lieb R., Pfister H., Hofler M., Wittchen H. U. (2002). What predicts incident use of cannabis and progression to abuse and dependence? A 4-year prospective examination of risk factors in a community sample of adolescents and young adults. Drug and Alcohol Dependence.

[b55] Wiehe S. E., Garrison M. M., Christakis D. A., Ebel B. E., Rivara F. P. (2005). A systematic review of school-based smoking prevention trials with long-term follow-up. Journal of Adolescent Health.

[b56] Williams R. J., Chang S. Y. (2000). A comprehensive and comparative review of adolescent substance abuse treatment outcome. Clinical Psychology-Science and Practice.

[b57] Windle M., Wiesner M. (2004). Trajectories of marijuana use from adolescence to young adulthood: Predictors and outcomes. Development and Psychopathology.

